# Editorial

**Published:** 1876-04

**Authors:** 


### Editorial.

### THE RUBBER COMPANY AGAIN.

We give below a letter from Mr. E. L. Hamilton the agent of the Celluloid Co. This letter is quite explicit in its statements, which seems to be what the profession want just now. We are in receipt of letters daily asking for information in reference to the operations and demands of the Dental Vulcanite Co.

This letter will answer some of these questions very explicity.

NEW YORK, Feb, 22d, 1876,
120 BROADWAY

DEAR SIR:

In answer to your letter on the subject of the suit brought by the Goodyear Dental Vulcanite Co. ,against Jared Kibbee of Port Huron, Michigan, the Celluloid Manufacturing Co., desire to say that while it is desirous and willing to defend any dentist against a charge of infringement of the Cummings' Patent by the use of Celluloid, yet it was impossible for that company to undertake such defense in Mr. Kibbee's case or in any case similarly situated. A plain statement of a few of the facts in that case will satisfy you of the truth of this. Mr. Kibbee, it seems, was sued, and a decree by default was obtained against him by the Vulcanite Co. A reference was then made to the Master appointed by the Court to fix the amount of damage which Mr. Kibbee was to pay, when Mr. Kibbee was summoned before the Master he entered into a stipulation with Mr. Bacon to allow a whole lot of testimony to be admitted in the case, which had been printed, and consisted of about three hundred pages, besides other testimony. Then the Master made his report finding that Mr. Kibbee should pay so much money. After the master had made his report, after a judgment, *pro confesso*, had been entered against Mr. Kibbee, and after Mr. Bacon had got in all the testimony in the case which he wanted, and after the time had gone by for putting in any further evidence, Mr. Kibbee for the first time gives the Celluloid Co., notice of the suit,—of what use was the notice at that stage of the game? The Celluloid Co., can not defend its customers unless it is allowed to do it by counsel selected by itself and who are acquainted with the defense and the facts. Mr. Kibbee, and every other dentist onght to know, that if Mr. Bacon is permitted to *make the facts* in the case and is allowed to introduce in a case just such facts or statements as he pleases, he can succeed as a matter of course. If the dentist sued will only permit him to furnish the facts he could easily obtain a decree of the court that a *gold plate* was an infringement of the Cumming's Patent! I do not know how Mr. Kibbee came to concede away his rights. The Celluloid Co., will gladly undertake the defense of every dentist who has used Celluloid solely, if the Company is notified at the outset and has control of the case from the beginning. That Company has tried its best in the past to have the question between Celluloid and Vulcanite decided finally. They thought they had succeded in getting the Vulcanite Co., up to the ring in the Flagg case, but Mr. Bacon backed out as soon as he saw defeat was certain, and has adopted the tactics, so familiar to him, of avoiding a real opponent, viz. the Celluloid Co., and seeking in out of the way places an opponent who knows nothing about the merits, upon whom he can easily impose. The Celluloid Co., in order to prevent Mr. Bacon and his Company from continuing such threats and from harrassing dentists, has brought a suit against the Goodyear Dental Vulcanite Co., in the Circuit Court of the United States in this city and has applied for an injunction to prevent such threats and suits against the customers of the Celluloid Co, The motion for an injunction has been set down for the 29th inst and if we can possibly force the motion in on that day we shall do so; but as Mr, Bacon's tactics are to avoid a decision we look for an application for delay from him, I trust this explanation will be satisfactory to the dentists and will show that our Company is doing all in its power to protect the dentists, Yours truly,

E. LUTHER HAMILTON,
For Celluloid Manufacturing Co.

### MISSISSIPPI VALLEY DENTAL SOCIETY

Held its thirty-second annual meeting on the 1st, 2d and 3d of March.

There was present a large number of its members and others of the profession.

The programme presented by the Executive Committee was a very good one, which called forth some good papers and very interesting discussions.

The meeting upon the whole was one of the best we have ever attended, and was greatly enjoyed by all present.

The age of this society seems to impart strength and energy to it. It never fritters away the time of its sessions on unprofitable subjects, which can hardly be said of some societies we know of.

### SOUTHERN DENTAL ASSOCIATION.

The eighth Annual meeting of the Southern Dental Association will be held in the city of Nashville, Tenn., April 11, 1876, at 10 o'clock a. m. All members of the dental profession, in good standing, are invited to attend.

JAS. F. THOMPSON, Sec.
Southern Dental Association

### COMMENCEMENT,

The annual commencement exercises of the Ohio College of Dental Surgery were held in the College on the evening of March 2d, 1876, in the presence of the Faculty, the Board of Trustees and a large assemblage of the profession and friends of the Institution. An address was delivered by the President of the Board of Trustees, and the annual address by Dr. W. H. Goddard of Louisville, Ky., and the response on behalf of the class by J. C. Kern. The degrees were conferred by the President on the following gentlemen, each of whom had sustained a satisfactory examination. and presented a thesis upon the subjects indicated, viz:

J. R. Bell, Ohio, Treatment of Exposed Pulps.

E. G. Betty, Ohio, Opium.
A. T. Good, Ohio, Dentistry, its relations to other Sciences
N. S. Hoff, Ohio, Development and Eruption of Deciduous Teeth.
W. R. Hale, Ohio, Causes of Decay.
C. I. Keely, Ohio, Filling Teeth.
W. D. Kempton, M. D., Ohio, Inflammation.
E. W. Poole, Ohio, Fifth Nerve.
C. F. Porter, Ohio, Digestion.
A. Taft, Ohio, Continuous Gum Dentures.
J. B. Kidd, Kentucky, Effects of First Dentition.
C. E. Canine, M. D., Kentucky, Nitrous Oxide.
E. H. Lathrop, California, Anæsthesia.
J. S. Clements, Wisconsin, Deposits on the Teeth.
J. G. Reid, Indiana, Diseases Caused by Decayed Teeth.
T. C. Kern, Nebraska. Deterioration of Teeth.

### A GOOD NAME.

Editor Register:

Would it not be a proper tribute to Dr, Barnum and also a great convenience to the profession if we should term the coffer-dam material *Barnum-rubber*, and call the pieces cut for use *Barnum's?*

There are so many, and such very obvious reasons for an affirmative answer to the proposition, that it seems needless to occupy more space in your journal than may be neccesary to thus put the question with the confident expectation of an echoing "aye" from every intelligent, honorable and appreciative dentist,

W. S. H.

### ILLINOIS DENTAL SOCIETY.

The Twelfth Annual Meeting of the Illinois State Dental Society will convene at Galesburg on Tuesday, May 9th, 1876.

A profitable and pleasant gathering, and large attendance is confidently expected.

All reputable dentists are cordially invited to be present.

### EXAMINATIONS.

We have so often been asked, "What is the course of the examinations to which candidates for graduation in the Ohio Dental College are subjected?" that we have concluded to give the questions in full to which the students in the recent examinations were required to give full written answers. In this instance there were no oral examinations.

The written answers were so satisfactory that oral examination was regarded as unnecessary. We do not present these questions as indicating a very rigid examination, still it is proper to say that no member of the class had any intimation or idea as to what the questions were till he was called upon to answer them, that being done in the presence of the Professors of the respective branches, and that without reference or consultation. The time occupied in the examination was over fifteen hours, seventy-five per cent of correct answers were required to pass the candidates for graduation.

In addition, each canditate was required to present a satisfactory artificial denture, upon *metallic* plate; and nearly all of these were continuous gum; and also, each one a case of practical treatment and operations in the way of filling in the mouth. These cases passed the scrutiny of the teachers and others who chose to see them. They were pronounced entirely satisfactory.

Now we have made these statements that the profession may have a better, fuller and clearer insight as to the course pursued in this Institution.

We give the questions in the order, and exactly as they were given to the students.

LIST OF QUESTIONS.

For the examination of the class for graduation, in the Ohio Dental College, March 2d, 1876.

ANATOMY.

1. Name all the bones of the face which are covered with mucus membrane?

2. Describe the upper jaw bone?

3. Describe the buccal cavity?

4. Name the muscles attached to the lower jaw?

5. Name the muscles connecting the head and thorax?

6. Give the origin and course of the artery which supplies the teeth of both jaws.

7. Name all the parts receiving nerves from both roots of the 5th Pair.

8. What would be the effect of Paralysis of the right facial nerve?

9. What organs contained in the thorax?

10. What organs contained in the abodmen?

PHYSIOLOGY.

1. Give the quantity, some general characters and anatomical elements of the blood, and coagulating principle?

2. Give the course of the blood from left ventricle, back to the same, and the various openings in the heart.

3. What property of the small vessels causes the blood to flow in a continuous stream?

4. What effect has respiration on the atmosphere?

5. Give some articles of nitrogenized food; also of non-nitrogenized.

6. What glands furnish saliva?

7. What takes place in the stomach on the entrance of food?

8. What takes place in the small intestine on the entrance of chyme?

9. What is the action of pancreatic juice?

10. How does absorption take place?

### PATHOLOGY AND MATERIA MEDICA.

1. What do you understand by immediate and remote effects?

2. Can the same remedy have immediate and remote effects? If so give an example.

3. Give two laxative and two hydragogue cathartics, and their doses.

4. How would you diagnose between poisoning by opium and belladonna, and what would be the treatment in each case?

5. Give three preparations of opium and their doses.

6. How is nitrate of silver made, and give its external uses.

7. Give the symptoms of inflammatory fever.

8. How would you diagnose an acute from a chronic inflammation, and give external and constitutional symptoms of each.

9. What is mortification and constitutional symptoms?

10. Give diagnosis and treatment of an abscess in the antrum.

### EXAMINATION IN CHEMISTRY.

1. Define an element.

2. Describe the process of ordinary combustion, the principle involved, and the products.

3. Upon what power of attraction does chlorine and some of its compounds act as a bleaching agent?

4. Describe the action of Hydrochloric acid on Dentine.

5. Give in symbolic equation the re-action between Nitric acid and Silver, and the change produced by adding to the compound a soluble chloride.

6. Name the element which appears to be an essential constituent of all acids.

7. Define the equivalency of an element; and also define a saturated compound.

8. Name two Perissad elements, also two Artiad elements.

9. Define the "Law of even numbers."

10. What is the relative density of H. O. Cl. and $N_2O$.

11. Define a compound radical, and name one of either equivalent or trivalent equivalency.

### OPERATIVE DENTISTRY.

1. What are the functions of the teeth?

2. Enumerate the diseases that should come to the special care of the dentist.

3. How is decay of the teeth produced?

4. Does *nature* ever arrest decay of the teeth? If so, how?

5. What hygienic treatment will best prevent the teeth from decay?

6. Give the pecularities of hyper-sensitiveness of the dentine.

7. What variations of this affection are found?

8. What is its treatment?

9. Why does persistent pain not attend exalted sensitiveness of dentine?

10. What is dental periostitis? and in what respect is it peculiar and what is the treatment?

11. What is suppuration?

12. What is ulceration?

13. What is the difference between the process of absorption as exhibited in living organic tissue and by inorganic bodies?

14. By what means is lost tissue restored?

15. Describe the process of granulation?

16. What constitutes a hæmorrhagic diathesis?

17. What is the treatment for aveolar hæmorrhage?

18. Give a list of Therapeutic agents, valuable and practicable for the purpose of a dentist in his general practice.

19. Give the treatment of exposed pulps.

### CLINICAL DENTISTRY.

1. Give time and order of the eruption of the permanent teeth.

2. Requisite qualities of a material for filling teeth.

3. In filling teeth what things are necessary to success?

4. Describe operation of filling a tooth with an exposed pulp, where there is no inflammation.

5. Describe operation and give treatment when there is inflammation of the pulp.

6. Give the successive steps in the formation of alveolar abscess.

7. Give treatment of alveolar abscess.

### MECHANICAL DENTISTRY.

1. How would you extract a superior cuspid broken off one eighth of an inch above edge of aveoli?

2. How soon after extraction would it be proper to insert a set of artificial teeth?

3. What are the principles to be observed in articulating full upper artificial sets of teeth to lower natural ones? also full upper and lower.

4. What are the requirements for a base for artificial teeth and what base fills that requirement in the highest degree?

5. What method of mounting teeth on gold plate is best and why?

6. What are the requiste properties for die and counter die and what metal is best?

7. Give successive steps in making full sets of continuous gum.

6. Describe the method of using the blow pipe and describe blow pipe flame.

9. How should an impression be taken when there are four or five scattering teeth in the jaw.

10. Give the method of restoring features marred from loss of teeth.

11. Give the method of obtaining a correct occlusion.

12. Describe the proper method of manipulating plaster and wax in taking impressions.

---

LAWRENCEBURG, IND., March 22, 1876.

J. TAFT, EDITOR:

Celluloid is almost certain to supersede all easily manipulated bases for artificial substitutes.

And if this can be truly said of it in its present condition, it will be very apt to answer all purposes in mechanical dentistry with the improvements of the future. Since writing you last about this base, I have been carefully experimenting and observing the effects of usage on the base and I can say that it is the nearest to what has long been needed that has yet been brought before the profession.

Some say if the camphor could be substituted with a mineral ingredient it would be preferable. I think that perhaps if some mineral substance was added for which the base has an affinity thereby making it stiff without interfering with its manipulation it would be an advantage and I fully believe this desirable improvement will be made in time. I think the antiseptic qualities of the camphor are desirable as almost all persons who are so careless as to neglect and lose the natural teeth, will and do treat artificial substitutes carelessly.

Camphor is a powerful antispectic and will very nicely counteract the offensive effects of oral secretions, while its presence causes no inconvenience whatever. My patients are delighted with its neat and cleanly appearance. While the most severe test, as to making a substitute and letting it remain in the drawer weeks before applying it has shown no warping or shrinking. I think the best way to work it is by "dry heat." In this the utmost disregard of directions, has shown how much abuse the stuff is capable of enduring.

I use a "Heindsman's heater" after trying the steam apparatus, and feel convinced that with dry heat is the right way to work it. The heating apparatus sold with the oven is too slow and the legs of the stove or oven are too short. I remedied this by setting the oven on three bricks that were placed edgewise and applying heat from Hull's Gasoline Burner. This though a noisy and odorous instrument means business when you want a quick and high temperature, bringing up to 280° and 380° in from 15 to 25 minutes; when all you have to do is to take away the fire and turn down the press, taking a peep occasionaly or taking out the flask opening and cutting off excess if thought best, and if in a hurry place in a White flask clamp and set in cold water. The whole time need not exceed an hour at most. I prefer to let the case cool in the oven if it does take longer, though I have not noticed any particular difference in slow or rapid cooling, but I am sure that time is saved and no harm done by the most rapid heating possible. Always before heating saturate the plaster floor of the oven with water. Let the incredulous try and see for themselves, it will cost but little and be sure to repay well, and I think they will join with me in voting thanks to the inventors of this base.

S. E. H.

### SALICYLIC ACID.

In a recent communication Dr. Rawls says: "Try Salicylic acid macerated into a thin paste with oil of cloves for treating pulpless teeth. Also for placing between an exposed pulp and the capping." He also says "try tincture of Tolu in canals of teeth through which there is a chronic and offensive discharge. It is one of the best disinfectants for such cases I have found."

We have made but very limited use of either of these preparations, but so far they have given good, results and are certainly worth a trial by every one.

### FLOWERS.

Vick's Floral Guide for 1876 comes to us ladened with interesting matter. It is replete with information, and no one who cares for the culture of flowers can afford to be without it.

We have for several years been supplied with seeds and bulbs from Vick's, and can say they have always given the most entire satisfaction, etc. We would recommend them to the readers of the REGISTER.

### OHIO DENTAL COLLEGE FACULTY CHANGES.

At the close of the recent session of the Ohio College of Dental Surgery, Prof. J. I. Taylor resigned the position of Professor of Clinical Dentistry, which he has held for ten years, the last of which was in conjunction with Dr. H. M. Reid, who will have full charge of this department for the present year

Prof. Taylor was a popular teacher.

Two special chairs were established, viz:

Diseases of women and children with referance to the dental organs. Geo. Watt, M. D., D. D. S.

Oral Surgery. F. H. Rehwinkel, M. D., D. D. S.

Both of these gentlemen are well known to the profession throughout the country, both eminently qualified for the positions to which they have been assigned. All the friends of the Institution will feel highly gratified for these appointments.

